# Seasonal Expression of Avian and Mammalian Daily Torpor and Hibernation: Not a Simple Summer-Winter Affair^†^

**DOI:** 10.3389/fphys.2020.00436

**Published:** 2020-05-20

**Authors:** Fritz Geiser

**Affiliations:** Centre for Behavioural and Physiological Ecology, Zoology CO2, University of New England, Armidale, NSW, Australia

**Keywords:** daily torpor, energy expenditure, heterothermy, hibernation, reproduction, season, torpor cost-benefit

## Abstract

Daily torpor and hibernation (multiday torpor) are the most efficient means for energy conservation in endothermic birds and mammals and are used by many small species to deal with a number of challenges. These include seasonal adverse environmental conditions and low food/water availability, periods of high energetic demands, but also reduced foraging options because of high predation pressure. Because such challenges differ among regions, habitats and food consumed by animals, the seasonal expression of torpor also varies, but the seasonality of torpor is often not as clear-cut as is commonly assumed and differs between hibernators and daily heterotherms expressing daily torpor exclusively. Hibernation is found in mammals from all three subclasses from the arctic to the tropics, but is known for only one bird. Several hibernators can hibernate for an entire year or express torpor throughout the year (8% of species) and more hibernate from late summer to spring (14%). The most typical hibernation season is the cold season from fall to spring (48%), whereas hibernation is rarely restricted to winter (6%). In hibernators, torpor expression changes significantly with season, with strong seasonality mainly found in the sciurid and cricetid rodents, but seasonality is less pronounced in the marsupials, bats and dormice. Daily torpor is diverse in both mammals and birds, typically is not as seasonal as hibernation and torpor expression does not change significantly with season. Torpor in spring/summer has several selective advantages including: energy and water conservation, facilitation of reproduction or growth during development with limited resources, or minimisation of foraging and thus exposure to predators. When torpor is expressed in spring/summer it is usually not as deep and long as in winter, because of higher ambient temperatures, but also due to seasonal functional plasticity. Unlike many other species, subtropical nectarivorous blossom-bats and desert spiny mice use more frequent and pronounced torpor in summer than in winter, which is related to seasonal availability of nectar or water. Thus, seasonal use of torpor is complex and differs among species and habitats.

## Introduction

The climate and weather of most geographical regions change substantially with season. This reflects to a large extent the yearly cycle of the rotating earth with its tilted axis around the sun, the distance from the equator, the elevation of the terrain, but also a number of specific local environmental factors. The resulting most obvious physical changes of seasonal environmental variables include ambient temperature (*T*_a_) and day-length, but often also a change in precipitation or wind speed. The change in *T*_a_ is crucial to many organisms, as it strongly affects their environment and their bodily functions, such as energy expenditure, thermoregulation, locomotion, reproduction and growth.

Especially endothermic (capable of producing substantial amounts of heat internally) birds and mammals are strongly, but indirectly, affected by *T*_a_ (Withers et al., 2016). Endotherms can regulate a high and constant body temperature (*T*_b_) over a wide range of *T*_a_ to a large extent by fine adjustments of heat production below the thermo-neutral zone (TNZ) and heat loss within and above the TNZ. In the TNZ the metabolic rate (MR) in normothermic (constant high “normal” *T*_b_) and resting endotherms can be minimal or “basal” (BMR). Above the TNZ cooling of the body is accomplished by evaporation of water, which is facilitated by sweating, increased ventilation or postural changes, and together with an increase in *T*_b_ results in an increase in MR. Below the TNZ metabolic rate is inversely related to *T*_a_, because heat loss is a function of the *T*_b_–*T*_a_ differential. To achieve this, the animals must produce large amounts of internal heat by shivering or non-shivering thermogenesis (Nowack et al., 2017a) to replace the heat lost from the body to the environment (Withers et al., 2016). As *T*_a_ is usually lower at night than during the daytime, mammals, most of which are nocturnal, are faced with an extra challenge, but to some extent can use heat generated by activity for thermoregulation. In contrast, most birds are diurnal and have to produce sufficient heat while resting during the night if they are to remain normothermic, although some of the birds to be discussed here are also nocturnal.

The majority of birds and mammals are small and because surface area and body mass or body volume are inversely related, heat loss is also strongly affected by body size (Withers et al., 2016). This can be problematic for small birds and mammals because compensation for heat loss by internal heat production is energetically expensive and requires the intake of large amounts of food to fuel the high metabolism. The time of year that is of special concern in many, but not all, regions is of course winter when *T*_a_ is low together with a low availability of food. To a large extent because of such energetic challenges many species that can fly (birds and bats) and can cover large distances fast and energetically cheaply (Tucker, 1975) avoid these conditions and migrate to more benign areas often over long distances. Small non-volant species cannot move over long distances because their locomotion is slow and energetically expensive (Tucker, 1975). Therefore sedentary species have to deal with thermal conditions and food availability in or near their usual home range by using other behavioral and physiological approaches instead (Körtner et al., 2000).

Many small endothermic mammals and birds are therefore not permanently homeothermic (have a constant *T*_b_), but rather are heterothermic (have a fluctuating *T*_b_) and use torpor for energy conservation, most typically during times of food shortage and/or cold exposure (Kayser, 1961; Dawson and Hudson, 1970; Lyman et al., 1982; Reinertsen, 1983; Boyer and Barnes, 1999; McKechnie and Lovegrove, 2002; Schleucher, 2004). However, torpor has many other selective advantages including water conservation, enabling reproduction and development, dealing with storms, fires, heat waves, floods or increased predator presence (Schmid and Speakman, 2009; Nowack et al., 2017b, 2020; Barak et al., 2019; Renninger et al., 2020). Torpor is characterized by substantial and controlled reductions in MR (often by 50–95%) and *T*_b_ (often by ∼5–35°C) and may last for a part of the day (daily torpor in the daily heterotherms), or for a number of days up to weeks (hibernation or multiday torpor in the hibernators), but multiday torpor is interrupted by periodic arousal to normothermic *T*_b_ in most. Daily torpor and hibernation in most heterotherms differ both ecologically and functionally. Hibernators often show extensive fattening before the torpor period, by ∼35% in dormice (*G. glis*; Fietz et al., 2003; Lebl et al., 2011; Bieber et al., 2014) or fewer store food (Humphries et al., 2003) and many species show no or only limited foraging during the hibernation season. Hibernators reduce *T*_b_ from ∼38 to ∼5°C (many to between 0 and 5°C) and the torpor MR (TMR) to ∼5% of BMR on average (Ruf and Geiser, 2015). In daily heterotherms, the *T*_b_ falls to ∼18°C and the TMR is ∼30% of the BMR on average (Ruf and Geiser, 2015; Shankar et al., 2018). Daily heterotherms may lose body mass or fatten only little before the torpor season and torpor is interrupted by periodic, often daily, foraging. Importantly, even during the state of torpor when the TMR is at a minimum, animals remain endothermic and can control their *T*_b_ above a critical minimum (Hainsworth and Wolf, 1970; Heller and Hammel, 1972), likely to avoid tissue or organ damage that seems to occur well above 0°C in many species, and, in species exposed to sub-zero *T*_a_s, to avoid freezing (Barnes, 1989).

**FIGURE 1:**
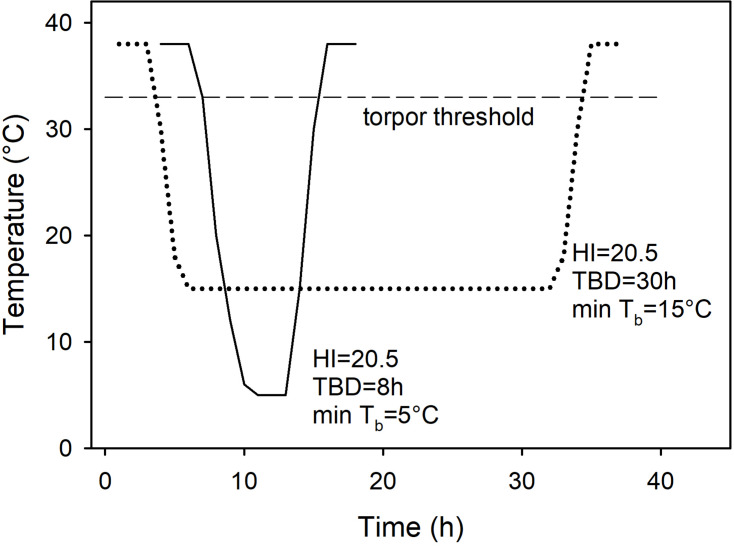
Two constructed, but realistic bouts of torpor, one deep and brief (solid line), the other shallow and long (dotted line) showing the change of body temperature over time and the respective heterothermy index (HI). Although the torpor bouts differ enormously with regard to the minimum *T*_b_ and the torpor bout duration (TBD), the HI is identical. Also shown is the torpor threshold (broken line), in this example at *T*_b_ = 33°C because the normothermic resting *T*_b_ = 38°C.

As it is sometimes claimed that there are no distinct patterns of torpor, but rather a continuum of variables when comparisons are based on *T*_b_ measures such as the ‘heterothermy index’ (e.g., Boyles et al., 2013), I attempted to use this approach initially for analyzing the data. However, since the heterothermy index failed to differentiate between deep, short and long, shallow torpor bouts resulting in the same heterothermy index for entirely different patterns of torpor (Figure 1), this method was not further pursued and the traditional approach using physiological minima or maxima expressed by a species was used for classification and analyses instead (Brigham et al., 2011; Ruf and Geiser, 2015).

Both daily torpor and hibernation, as stated above, are typically interrupted by energetically costly periodic arousals using endogenous heat production, and maintenance of normothermic *T*_b_ for periods usually lasting for several hours (Boyer and Barnes, 1999; McKechnie and Lovegrove, 2002; Ruf and Geiser, 2015). However, despite periodic arousals and the long duration of the hibernation season for about two-thirds of a year, energy expenditure during the hibernation season was only 13-17% of the annual energy expenditure in ground squirrels (*Callospermophilus* syn. *Spermophilus saturatus*) (Kenagy et al., 1989). In pygmy-possums (*Cercartetus nanus*) energy expenditure during a one-year hibernation season in captivity was only ∼4% of the predicted field metabolic rate (Geiser, 2007).

Whereas daily heterotherms often use the normothermic periods between torpor bouts for foraging and feeding (Körtner et al., 2008) in hibernators the physiological requirement of periodic arousals to normothermic *T*_b_ is still not fully understood. However, as outlined below, it is likely linked to restorative functions to counteract some dysfunction that occurred during torpor (Willis, 1982; Geiser et al., 1990; Daan et al., 1991; Prendergast et al., 2002). Periodic rewarming from hibernation seems to be related to the usually low *T*_b_ experienced during deep torpor because in a few species periodic rewarming does not occur during hibernation at high *T*_b_ (Lovegrove et al., 2014; but see Liu and Karasov, 2011), or periodic rewarming is partly or entirely passive (Dausmann and Warnecke, 2016), which seems to suffice. With regard to migration, although it is used to avoid adverse conditions in many flying species, migration does not preclude torpor as it can have an important role during migration stopovers in birds as well as in bats to save energy and maximize energy availability for the next flight (Carpenter and Hixon, 1988; Hiebert, 1993; Wojciechowski and Pinshow, 2009; McGuire et al., 2014).

Torpor is extremely diverse and occurs in all mammalian subclasses and in at least ten avian orders (McKechnie and Lovegrove, 2002; Ruf and Geiser, 2015). Torpor also has been described in all geographic regions of the world and these differ substantially with regard to seasonal challenges. Whereas temperate and high latitude/altitude regions are typically characterised by warm *T*_a_s in summer and often high primary productivity, *T*_a_s in winter are low resulting in little or no primary productivity, tropical areas may remain rather warm in winter, but often show strong seasonal changes in rainfall with almost all precipitation in summer and none in winter. In subtropical areas the high summer heat may limit plant productivity and, for example nectar production, can be much higher during the mild winters (Ford, 1989). In deserts *T*_a_s are often too hot and/or precipitation too low in summer for significant plant growth, whereas winters can be rather mild during the day at least in deserts not too far from the equator, as for example the Australian deserts. The seasonal change in photoperiod, a reliable environmental signal for seasonal change in physiology, also differs enormously between high and low latitudes. Such regional differences are reflected in the seasonal expression of torpor.

My review aims to summarize the different seasonal patterns of torpor use of endotherms and relate them to their environment and reproduction. Other aspects of the seasonal change in torpor expression have been reviewed recently with regard to seasonal energy use (Kenagy, 1989) circadian and circannual rhythms (Körtner and Geiser, 2000b) or its neural and endocrine control (Jastroch et al., 2016). I tried to use data on free-ranging animals and animals kept under natural photoperiod and *T*_a_ fluctuations or captured in the wild, but data on seasonal torpor under laboratory conditions, if they seemed relevant, are also reported. Overall, much fewer quantitative data are available for summer than for winter. Known torpor occurrence for the four seasons in hibernators and daily heterotherms were analyzed using Chi-Square tests. Seasons are defined as spring (March to May), summer (June to August), autumn (September to November), and winter (December to February) for the northern hemisphere and the reverse for the southern hemisphere. The data on seasonal torpor expression are also used to examine hypotheses in relation to the widely assumed clearly different seasonal summer and winter phenotypes for heterotherms in general (e.g., Williams et al., 2005) and whether the observed seasonal torpor expression can be explained by or supports the cost-benefit approach outlined by Humphries et al. (2003).

Sections are generally summarized under different orders or sub-classes, but since rodents were the first group that was examined in detail with regard to seasonal hibernation in the field, they are covered first. The tables are presented in the usual taxonomic order. As most quantifications of torpor were based on *T*_b_ measures, a reduction of *T*_b_ by >5°C below the normothermic resting *T*_b_ was used to define torpor (example shown in Figure 1); the time when *T*_b_ remained >5°C below resting *T*_b_ was defined as torpor bout duration (TBD) (Ruf and Geiser, 2015). Hibernators are defined as species that can express multiday torpor of >2 days, whereas daily heterotherms are defined as species expressing daily torpor exclusively under all thermal, environmental and nutritional conditions. “Shallow” torpor describes a reduction of *T*_b_ by 5–10°C below normothermic resting *T*_b_. When only MR measures were available, a reduction of MR by >25% below the RMR at the same *T*_a_ was used to define torpor (Hudson and Scott, 1979).

## Hibernation

**FIGURE 2:**
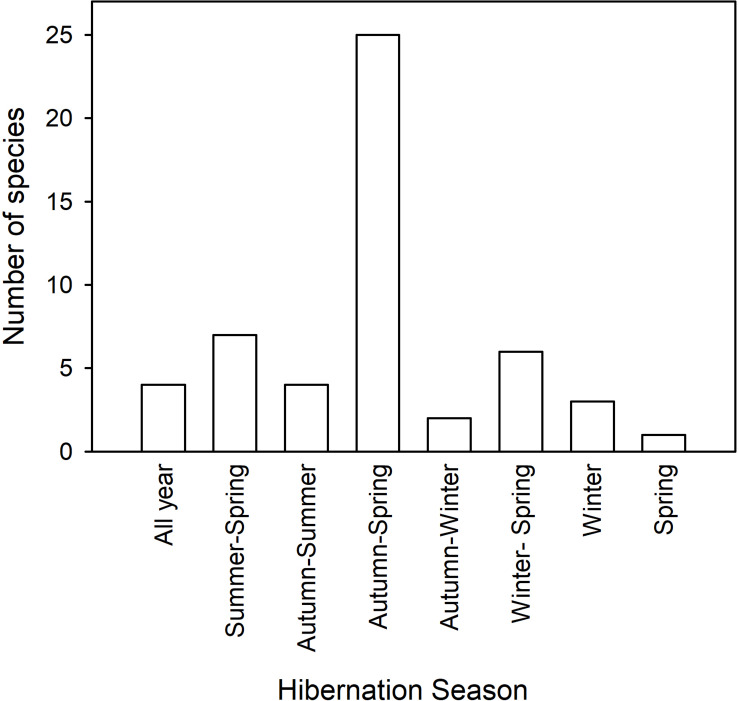
The duration of the hibernation season and the number of species expressing multiday torpor during each season is shown. The data and references are listed in Table 1.

It has been known for centuries that some mammals disappear in winter and that they hibernate during this time. Much of the original work was done in northern Europe or America with some quantitative work appearing in the early eighteen-hundreds Hall (1832) or even before (see Kayser, 1961; Mrosovsky, 1971; Lyman et al., 1982). Hibernation is especially obvious in diurnal sciurid rodents, such as ground squirrels, chipmunks and marmots, which disappear into burrows around autumn and re-appear in spring and their seasonal use of torpor can be quantified to a large extent by observation or trapping. It is therefore of little surprise that the widely held view of seasonal expression of torpor, or specifically hibernation (from Latin “hibernare” to spend the winter) is one of torpor use in late autumn, winter and early spring, unlike the rest of the year, which is supposed to be devoted to activity and reproduction. Obviously, the term hibernation has a seasonal connotation implying that it only occurs in winter, but, as we will see below, hibernation is only rarely restricted to winter and may in fact last for much of the year in some species and under certain circumstances (Figure 2).

### Yearlong Hibernation

The most extreme expression of hibernation is known for three unrelated mammals, phylogenetically separated for >120 Million years, the marsupial eastern pygmy-possum (*Cercartetus nanus*) from Australia and the dormice (*G. glis* and *Eliomys quercinus*), rodents from Eurasia (Figure 2 and Table 1). These hibernators do not only hibernate in winter, but, under laboratory conditions, can do so for an entire year. In captivity the pygmy-possums managed to hibernate at *T*_a_ 7°C for over 12 months, relying entirely on stored body fat for energy expenditure (Geiser, 2007). Edible dormice (*G. glis*) bought from a breeder in France hibernated in a Canadian cold room at *T*_a_ 5°C for up to a year, but since body mass cycles were investigated it appears that food was available and animals may have fed (Mrosovsky, 1977). In another study, captive non-reproductive *G. glis* hibernated for up to 11 months/year, and, as animals had access to food and were in good condition, the prolonged underground hibernation was interpreted as having evolved as means for predator avoidance in the wild (Bieber and Ruf, 2009). Free-ranging non-reproductive *G. glis* also can hibernate for up to 11 months (Hoelzl et al., 2015). Captive garden dormice (*E. quercinus*), also purchased from France, expressed torpor throughout the year when held in Holland at *T*_a_ 12°C, but the TBD was shorter in summer than in winter (Daan, 1973).

**TABLE 1:** The hibernation season when multiday torpor (>2 days) can be used by birds and mammals.

**All year**
Eastern pygmy possum, *Cercartetus nanus* (Geiser, 2007)
Long-eared bat, *Nyctophilus geoffroyi* (Turbill et al., 2003; Turbill and Geiser, 2008)
Edible dormouse, *Glis glis* (Mrosovsky, 1977; Hoelzl et al., 2015)
Garden dormouse, *Eliomys quercinus* (Daan, 1973)
**Summer to spring**
Echidna, *Tachyglossus aculeatus* (Grigg et al., 1989; Nicol and Andersen, 2007)
Anatolian ground squirrel, *Spermophilus xanthoprymnus* (Kart Gür et al., 2009)
Arctic ground squirrel, *Urocitellus/Spermophilus parryii* (Barnes, 1996)
Columbian ground squirrel, *Urocitellus*/*Spermophilus columbianus* (Young, 1990)
Richardson’s ground squirrel, *Urocitellus/Spermophilus richardsonii* (Wang, 1978)
Golden-mantled ground squirrel, *Callospermophilus/Spermophilus saturatus* (Barnes, 1996)
Woodchuck, *Marmota monax* (Zervanos et al., 2010)
**Autumn to summer**
Little brown bat, *Myotis lucifugus* (Jonasson and Willis, 2012; Johnson et al., 2019)
Brandt’s bat, *Myotis brandtii* (Podlutsky et al., 2005)
Hodgson’s bat, *Myotis formosus* (Kim et al., 2013)
Jumping mouse, *Zapus princeps* (Cranford, 1978)
**Autumn to spring**
Mountain pygmy-possum, *Burramys parvus* (Körtner and Geiser, 1998)
Fat-tailed dwarf lemur, *Cheirogaleus medius* (Dausmann, 2014)
Dwarf lemur, *Cheirogaleus crossleyi* (Blanco et al., 2013; Dausmann, 2014)
European hedgehog, *Erinaceus europaeus* (Walhovd, 1979)
Algerian hedgehog *Atelerix algericus* (Mouhoub-Sayah et al., 2008)
Greater horseshoe bat, *Rhinolophus ferrumequinum* (Park et al., 2000)
Big brown bat, *Eptesicus fuscus* (French, 1985)
Indiana bat, *Myotis sodalis* (Clawson et al., 1980)
Mouse-tailed bat, *Rhinopoma microphyllum* (Levin et al., 2015)
Mouse-tailed bat, *Rhinopoma cystops* (Levin et al., 2015)
Brown bear, *Ursos arctos* (Evans et al., 2016)
Yellow-pine chipmunk, *Tamias amoenus* (Barnes, 1996)
Townsend’s chipmunk, *Tamias townsendi* (Barnes, 1996)
Siberian chipmunk, *Eutamias sibiricus* (Kawamichi and Kawamichi, 1993)
Golden-mantled ground squirrel, *Callospermophilus/Spermophilus lateralis* (Healy et al., 2012)
Daurian ground squirrel, *Spermophilus dauricus* (Yang et al., 2011)
Thirteen-lined ground squirrel, *Ictidomys/Spermophilus tridecemlineatus* (Kisser and Goodwin, 2012)
Alpine marmot, *Marmota marmota* (Arnold, 1993)
Alaska marmot, *Marmota broweri* (Lee et al., 2009)
Yellow-bellied marmot, *Marmota flaviventris* (French, 1985)
Prairie dog, *Cynomys parvidens* (Lehmer and Biggins, 2005)
European hamster, *Cricetus cricetus* (Wassmer, 2004; Siutz et al., 2016)
Common or hazel dormouse, *Muscardinus avellanarius* (Pretzlaff and Dausmann, 2012)
Woolly dormouse, *Dryomys laniger* (Kart Gür et al., 2014)
Birch mouse, *Sicista betulina* (Eisentraut, 1956)
**Autumn to winter**
Armadillo, *Zaedyus pichiy* (Superina and Boily, 2007)
African hedgehog, *Atelerix frontalis* (Hallam and Mzilikazi, 2011)
**Winter to spring**
Pygmy slow loris, *Nycticebus pygmaeus* (Ruf et al., 2015)
European free-tailed bat, *Tadarida teniotis* (Arlettaz et al., 2000)
Formosan leaf-nosed bat, *Hipposideros terasensis* (Liu and Karasov, 2011)
Black bear, *Ursus americanus* (Toien et al., 2011)
Eastern chipmunk, *Tamias striatus* (Landry-Cuerrier et al., 2008)
Jerboa, *Jaculus orientalis* (El Ouezzani et al., 2011)
**Winter**
Poorwill, *Phalaenoptilus nuttallii* (Woods et al., 2019)
Western pygmy-possum, *Cercartetus concinnus*, field (Turner et al., 2012b)
European badger, *Meles meles* (Fowler and Racey, 1988).
**Spring**
Golden mole, *Amblysomus hottentottus longiceps* (Scantlebury et al., 2008)

*Only species for which long-term data are available are listed and the most pronounced pattern observed for each species is reported.*

It could be argued that yearlong hibernation or at least the yearlong use of torpor also occurs in long-eared bats (*Nyctophilus* spp.) and perhaps other bats as they can display multiday torpor throughout the year in the wild including during the period of reproduction (see below). In mouse-eared bats (*Myotis myotis*) torpor expression was largely independent of season but rather was affected by *T*_a_ (Wojciechowski et al., 2007). Moreover, in several species held in captivity (female marmots *Marmota flaviventris*, ground squirrels *Urocitellus* syn. *Spermophilus beldingi*, jumping mice *Zapus princeps*), hibernation could be artificially extended by continued cold exposure to >300 days (French, 1985). These findings suggest that hibernation for up to one year may be functionally possible in many hibernators.

### Seasonal Hibernation

### Mammals

### Rodents

A common seasonal pattern of hibernation in the northern hemisphere is torpor expression from late summer/autumn to spring. It occurs and has been investigated in detail in sciurid rodents such as ground squirrels, chipmunks and marmots (Sciuridae), dormice (Gliridae) and hibernating hamsters (Cricetidae).

The classic pattern of seasonal hibernation in free-ranging mammals was first quantified using temperature-telemetry for Richardson’s ground squirrels (*Urocitellus* syn. *Spermophilus richardsonii*) near Edmonton, Canada (Wang, 1978). The hibernation season in most individuals commenced in mid-July in adults, 2 months later in juveniles and was terminated by both groups in mid-March. Torpor in *U. richardsonii* was characterized by minimum *T*_b_s of ∼2°C and TBDs of 10–20 days in mid-winter in the wild, but shorter in captive individuals (Wang, 1978). More specific data, especially on sexual differences, have been provided for this species by Michener (1992) and confirm that in this ground squirrel hibernation in the wild lasts from summer until spring (Table 1). Similar patterns of seasonal torpor expression (Table 1) have been observed in golden-mantled ground squirrels (*C. lateralis* and *C. saturatus*) (Kenagy et al., 1989; Healy et al., 2012), Columbian ground squirrels (*Urocitellus* syn. *Spermophilus columbianus*) (Young, 1990) and Anatolian ground squirrels (*Spermophilus xanthoprymnus*) (Kart Gür et al., 2009). Hibernation from summer to spring is rather common and occurs in ∼14% of the species for which data are available (Figure 2 and Table 1).

Due to the far northern distribution of Arctic ground squirrels (*Urocitellus* syn. *Spermophilus parryii*), the hibernation season is also extremely long usually lasting from August/September to April (Barnes, 1996) and deep with minimum *T*_b_s as low as −2.9°C (Barnes, 1989; Richter et al., 2015). The beginning and end of the hibernation season differs between males and females, with males entering torpor later and emerging earlier than females to establish territories and get ready for mating (Barnes, 1996). Although the end of hibernation in March/April is inflexible in reproductive males, resulting in a potentially disastrous phenological mismatch during spring snow storms, non-reproductive males and reproductive females, after the end of the usual hibernation season, re-entered hibernation with short TBD of 1–6 days, emerged in May and thus delayed reproduction reducing the time available for growth of young and pre-hibernation fattening for the next winter (Williams et al., 2017).

In most hibernators (48% of species; Table 1 and Figure 2) including thirteen-lined ground squirrels (*Ictidomys* syn. *Spermophilus tridecemlineatus*) in Michigan and Daurian ground squirrels (*Spermophilus dauricus*) in northern China, the hibernation season lasts from autumn to spring (Yang et al., 2011; Kisser and Goodwin, 2012). Thus a hibernation season lasting for more than 6 months is most common. This is also the case for European alpine marmots (*Marmota marmota*). Alpine marmots show social hibernation, periodic rewarming is highly synchronized among individuals and the degree of synchrony affects mass loss during winter (Arnold, 1988, 1993; Ruf and Arnold, 2000). When juveniles were part of a hibernating group, territorial males commenced the rewarming process earlier than juveniles, which can use the heat generated by adults for partial passive rewarming. Woodchucks (*Marmota monax*) have a very wide distribution over North America and their hibernation season differs according to latitude (Zervanos et al., 2010). In Maine (∼44°N) woodchucks hibernated from July to April, in Pennsylvania (∼40°N) from November to March and in South Carolina (∼35°N) from December to March showing a strong phenotypic flexibility or selection among populations (Zervanos et al., 2010). During a severe drought in Pennsylvania, free-ranging *M. monax* entered short bouts of torpor in August with *T*_b_ fluctuating between ∼25 and 38°C when *T*_a_ ranged from 20 to 30°C; after rainfall some individuals remained normothermic, whereas others continued to exhibit torpor (Zervanos and Salsbury, 2003). With regard to elevation, although the hibernation season in prairie dogs (*Cynomys parvidens*), which are considered to be “facultative” hibernators, lasted from autumn to spring in high and mid-elevation populations, low elevation populations terminated hibernation already in late winter, when food became available (Lehmer and Biggins, 2005).

Unlike many other hibernators, European hamsters (*Cricetus cricetus*) store food rather than mainly fat for the hibernation season (Wassmer, 2004; Siutz et al., 2016). Consequently, the gut is not reduced but rather needs to be maintained during winter (Humphries et al., 2003; Tissier et al., 2019). Nevertheless, the hibernation season is similar to that of many other rodent hibernators lasting from autumn to spring (Figure 2), however, the TBD is somewhat shorter (∼5 days) than in many other species (TBD often 10–20 days), and the usual sexual differences in the hibernation season are reversed, with adult males hibernating for longer than females (Siutz et al., 2016).

Chipmunks are often considered to be intermediate between food-storing and fat-storing rodent hibernators, and, although it has been claimed they rely entirely on stored food during hibernation (Humphries et al., 2003), some can also store substantial amounts of fat. In free-ranging Siberian chipmunks (*Eutamias sibericus*) measured in Hokkaido, Japan, over 7 years, hibernation commenced first in adults in September/October followed by juveniles about 1 month later; spring emergence occurred around April in adult males and in May in females and the yearly variation in the timing of hibernation reflected snow cover (Kawamichi and Kawamichi, 1993). Mortality during hibernation for all age classes was low (3.7–5.7%) whereas during the active period mortality in adults was around 50% (Kawamichi and Kawamichi, 1993). In western chipmunks, such as yellow-pine (*Tamias amoenus*) and Townsend chipmunks (*Tamias townsendi*) the hibernation season in the wild in Washington, United States, was somewhat shorter than in sympatric ground squirrels and lasted from around October/November to March (Kenagy and Barnes, 1988), but they still fall in the group with the most common hibernation season (Figure 2 and Table 1). Hibernation in *T. amoenus* is rather predictable and, at least in the laboratory, is associated with substantial fattening (∼45% increase in body mass) in autumn, and animals eat little or nothing when hibernating during mid-winter despite availability of food (Geiser et al., 1990). On the other hand, in eastern chipmunks (*Tamias striatus*), studied in Quebec, torpor use differs from many other sciurids as its expression in winter even in nature can be rather variable to a large extent depending on food availability (Landry-Cuerrier et al., 2008). In good food years when many trees produced seeds, torpor in *T. striatus* was used in winter but was rather irregular and shallow (*T*_b_ often >10°C). Whereas in low-food years hibernation was characterized by a regular expression of a sequence of deep (*T*_b_<10°C) and multiday torpor bouts and lasted from ∼November/December to May (Landry-Cuerrier et al., 2008). The seasonal pattern observed in *T. striatus* occurs in only ∼11% of hibernators, including mainly species from mild climates and large bears (Table 1). Hudson (1978) extrapolated from data on *T. striatus* that chipmunks in general may differ from “classical” hibernators by having rather high minimum *T*_b_s of 5–7°C and because only some individuals expressed torpor in captivity. In contrast, *T. amoenus* can have very low minimum T_b_s during torpor (minimum regulated *T*_b_ −1.0°C, Geiser et al., 1994) and all individuals entered torpor in captivity, although the TBD generally was somewhat shorter (∼8 days) than in sympatric ground squirrels (*C. saturatus*, ∼11 days) in mid-winter (Geiser et al., 1990). Consequently, variables of torpor measured for *T. striatus* differ from many other hibernators in an interesting way, but because of that should not be considered representative of other sciurid rodents or hibernators in general.

Overall, available information suggests that hibernation in sciurid and cricetid hibernators is a strongly seasonal event, supporting the view of different seasonal phenotypes. Nevertheless, there is some flexibility in several species, especially in juveniles, females and non-reproductive males, which can extend the hibernation season when this is required.

In the dormouse family (Gliridae) hibernation is used by several species not covered above and in several dormice torpor expression is not highly seasonal. In addition to hibernation from autumn to spring (Pretzlaff and Dausmann, 2012), hazel dormice (*Muscardinus avellanarius*) frequently expressed torpor during summer, although torpor bouts were generally brief (Pretzlaff et al., 2014). Adult male *M. avellanarius* used torpor more frequently than females during the active season in summer and pregnant females used only shallow torpor, but females with litters and juveniles without mothers occasionally were observed in torpor (Juskaitis, 2005). Hibernation in captive wooly dormice (*Dryomys laniger*) lasted from October to April (Kart Gür et al., 2014). Other hibernating dormice include the African dormouse (*Graphiurus murinus*), which also may express torpor throughout the year since torpid animals were observed both in summer (Webb and Skinner, 1996) and winter (Mzilikazi et al., 2012).

In the dipodids (jerboas, jumping mice, birch mice) hibernation from winter to spring has been observed in the Egyptian jerboa, *Jaculus orientalis* (El Ouezzani et al., 2011). As mentioned above, jumping mice (*Zapus princeps*) hibernate in captivity for over 300 days (French, 1985), and just under 300 days (September to early July) at >2,000 m elevation in Utah in the field (Cranford, 1978). Birch mice (*Sicista betulina*) hibernate for 6–8 months (Eisentraut, 1956).

### Insectivores

Well-known hibernators in the insectivores (now Lipotyphla) are the hedgehogs. This includes the European hedgehog (*Erinaceus europaeus*), which has been investigated with regard to its hibernation physiology for decades (Kristoffersson and Soivio, 1964; Warnecke, 2017). Danish *E. europaeus*, kept in large outdoor pens, remained within their hibernacula continuously for up to 6 months and the hibernation season lasted from about October to April (Walhovd, 1979) the common seasonal pattern (Figure 2). Algerian hedgehogs, *Atelerix algericus*, held individually in a room with open windows near the Mediterranean Sea, commenced the hibernation season with short bouts of torpor in November, expressed long TBDs of 6–7 days in January/February, and ended the torpor season again with short bouts in March (Mouhoub-Sayah et al., 2008). The southern African hedgehog (*Atelerix frontalis*), held under semi-natural conditions in the Karoo, South Africa, hibernated from early May (Autumn) to late July (Winter) and this seasonal pattern is rather rare in the now available data (Figure 2). The minimum regulated *T*_b_ of *A. frontalis* was 1°C and TBD lasted for up to ∼5 days (Hallam and Mzilikazi, 2011).

### Bats

After the rodents (>2,000 species), bats are the second largest mammalian order with around 1,300 species and many, likely the vast majority, can use some form of torpor (Lyman, 1970; Stawski et al., 2014). To my knowledge data on hibernation in bats are currently restricted to the largely insectivorous “microbats”. There are no published data on multiday torpor in the largely frugivorous “megabats,” however, it is rumoured that some may be able to do it. Although many insectivorous bats are known to hibernate, detailed information on the hibernation season based on transmitter data is not as overabundant as in rodents. To some extent this is related to their small size, which creates technical limitations for tagging them with recording equipment. Transmitters or loggers must be small and therefore will not last for many months as is the case for larger devices used on medium to large hibernators. For these reasons, some bats were captured in winter (e.g., Arlettaz et al., 2000; Hope and Jones, 2012; Jonasson and Willis, 2012) so the exact beginning of the hibernation season remains unknown. Moreover, insectivorous bats are not easily kept in captivity for prolonged periods simply because of their food. However, some microbats are known to show extremely long TBDs in winter lasting up to 45 days as reliably measured by transmitters (Jonasson and Willis, 2012). Another complication with regard to the seasonality of torpor in bats is the lack of a clear distinction between the hibernation season and reproductive season as many bats use torpor during reproduction (Stawski et al., 2014). Bats hibernate under a vast variety of conditions (Webb et al., 1996; Turbill and Geiser, 2008; Meierhofer et al., 2019) and patterns of torpor differ widely among species and habitats. Bats have been reported to have very low minimum regulated T_b_s during torpor, often around 0°C or slightly below in cold regions, but even in tropical and subtropical areas minimum regulated *T*_b_s between 4 and 8°C have been measured (Geiser and Stawski, 2011; McKechnie and Mzilikazi, 2011).

It was already recognized over 170 years ago that small insectivorous bats or microbats display multiday torpor in winter and brief bouts of torpor in summer (Hall, 1832). In the pre-hibernation season long-eared bats (*Plecotus auritus*) selected low *T*_a_ of ∼10°C, in comparison to summer when they preferred thermo-neutral conditions, and entered torpor for ∼14 h/day as an energy sparing mechanism to enhance fat stores (Speakman and Rowland, 1999). Greater horseshoe bats (*Rhinolophus ferrumequinum*), roosting in caves in southern England, hibernated from mid-October to late May, the most common hibernation season (Figure 2), when torpor bouts ranged from ∼1.5 to 12 days (Park et al., 2000). Free-ranging Canadian long-eared myotis (*Myotis evotis*), in addition to hibernation in winter, use torpor on every day between May and August and even when reproductively active (Nagorsen and Brigham, 1993; Chruszcz and Barclay, 2002). Little brown bats (*Myotis lucifugus*) hibernate from about October/November to March/April and express torpor also in summer including during pregnancy and lactation with *T*_skin_ falling below 10°C (Jonasson and Willis, 2012; Johnson et al., 2019). Daubenton’s bat (*Myotis daubentoni*) hibernate (Ransome, 1990), and in late summer in central Germany reproductive females enter torpor mainly during post-lactation, whereas males use torpor frequently even during reproductive period in early summer (Dietz and Kalko, 2006). Pregnant female hoary bats (*Lasiurus cinereus*) entered prolonged torpor with *T*_skin_ as low as 5.5°C in southern Canada during inclement weather in late spring/early summer. This not only conserved energy, but delayed parturition during the cold spell and bats rewarmed when *T*_a_ increased to give birth under more favorable conditions for neonatal survival (Willis et al., 2006).

In warmer regions, Hodgson’s bats (*Myotis formosus*) hibernated in abandoned mines in southern Korea from October to June and interestingly maintained *T*_skin_ >11°C because of the high *T*_a_ (Kim et al., 2013). The rather large (∼60 g) Formosan leaf-nosed bat (*Hipposideros terasensis*) hibernated in abandoned tunnels in Central Taiwan from late December to early March (Liu and Karasov, 2011). Despite rather high *T*_skin_ of >20°C, TBD was up to 19 days, but periodic rewarming was observed. Subtropical fishing bats (*Myotis vivesi*) hibernated on desert islands in the Gulf of California and expressed torpor in summer when *T*_a_s were extremely hot (Salinas-Ramos et al., 2014). Mouse-tailed bats (*Rhinopoma microphillum* and *R. cystops*) fatten in August on winged ants, hibernate with *T*_skin_ of ∼23°C and partial arousals from late October for 5 months to early spring in geothermally heated caves in cliffs at the Sea of Galilee at *T*_a_ ∼20°C (Levin et al., 2015).

Most bats from cold-temperate regions hibernate in thermally stable hibernacula like caves, mines or cellars. However, some bats hibernate in trees. Long-eared bats, *Nyctophilus geoffroyi* and *N. gouldi*, hibernate during winter in trees under exfoliating bark facing the sun (2/3 of observations) or shallow tree cavities (1/3 of observations) in a cool-temperate area in south-eastern Australia (Turbill and Geiser, 2008). Despite partial passive rewarming each day, torpor bouts in mid-winter lasted for 5 days on average with a maximum of 15 days. Arousals were often brief <3 h, but on warm nights it appears that bats were foraging and feeding, explaining how they survive the winter without obvious pre-hibernation fattening. In a tropical area in winter when *T*_a_ ranged from 16.5 to 34.0°C, *N. geoffoyi* expressed short bouts of torpor on every day (Geiser et al., 2011). In a cool-temperate area in summer *N. geoffoyi* used short bouts of torpor on warm days on every day and on cool days TBDs up to 2 days were observed (Turbill et al., 2003). During the reproductive period in spring *N. geoffroyi* still used torpor when measured overnight in captivity at *T*_a_ 15°C (Turbill and Geiser, 2006). All males, pregnant females and lactating females entered torpor under these rather mild thermal condition, and during torpor, the minimum *T*_b_ was only ∼0.5°C above *T*_a_, and the minimum TMR was low and similar to that predicted for deep hibernators (Ruf and Geiser, 2015). As variables of torpor in *N. geoffoyi* and also *N. gouldi* did not differ among reproductive groups, it appears that some of the observed differences in torpor patterns in reproductive bats in the wild may be behavioral or ecological rather than physiological (Turbill and Geiser, 2006). For tree-roosting hibernating bats restricted to the tropics and subtropics, such as the northern long-eared bat *N. bifax*, torpor in a subtropical population was observed on 100% of days in winter and 85% of days in summer, TBD lasted for up to 5 days in winter and <1 day in summer, and body mass was indistinguishable between seasons (Stawski and Geiser, 2010b). Moreover, subtropical *N. bifax* expressed more torpor in summer when they were fat than lean suggesting that when they can energetically afford it, they use torpor to minimize foraging requirements and thus exposure to predators (Stawski and Geiser, 2010a).

As some bats enter torpor both in summer and winter and even during reproduction, the question arises whether there is a true seasonal change in physiology. As stated above, the minimum TMR of reproductive *Nyctophilus* bats was as predicted for deep hibernators of the same BM, and only about 10% of that in daily heterotherms on average (Ruf and Geiser, 2015). Similarly, non-reproductive *Nyctophilus* bats, from temperate, subtropical and tropical habitats, did not significantly change TMR with season, their TMR was as predicted for hibernators and was reached within ∼4 h when bats were thermo-conforming during torpor entry, and cardiac electrophysiology and heart rates of torpid bats also remained unchanged across summer, autumn and winter (Geiser and Brigham, 2000; Stawski and Geiser, 2011; Currie, 2015, 2018). As TBD is strongly temperature-dependent, and torpor bouts above the minimum regulated *T*_b_ are reversely related to *T*_a_ (Twente and Twente, 1965; Geiser and Kenagy, 1988), the short and shallow torpor bouts expressed by many microbats at high *T*_a_ in summer seem to reflect mainly ambient thermal conditions, rather than a change in physiology. Moreover, long bouts of torpor are expressed in summer in bats and also pygmy-possums (see below) when they are exposed to low *T*_a_. Thus, from a thermal energetics point of view, the brief and shallow torpor bouts in summer seem to be short bouts of hibernation, rather than daily torpor as expressed by daily heterotherms, which have much higher TMRs (Geiser and Brigham, 2000; Stawski and Geiser, 2011) and the same is the case for some marsupial pygmy-possums (see below). These observations suggests that at least in some hibernating bats, there is little or no seasonal change in the physiology of torpor, and do not provide support for the view of strong seasonal phenotypes in hibernators in general.

### Primates

Seasonal hibernation has been observed in several species of dwarf lemurs of Madagascar during the cool dry winter (Dausmann, 2014; Dausmann and Warnecke, 2016). The best-studied species is the fat-tailed dwarf lemur (*Cheirogaleus medius*). Although it hibernates from autumn to spring, its pattern of hibernation is rather unusual. Individuals that hibernate in poorly insulated tree hollows, show strong daily fluctuations of *T*_b_ with *T*_a_ and do not show periodic endogenous arousals. In contrast, *C. medius* that hibernate in well-insulated tree hollows show periodic arousals about once/week (Dausmann and Warnecke, 2016), similar to *C. crossleyi*, hibernating underground (Blanco et al., 2013). However, primate hibernation is not limited to Madagascar. Pygmy slow loris (*Nycticebus pygmaeus*) hibernate from winter to spring in tropical Vietnam (Ruf et al., 2015) albeit their TBDs are brief (up to ∼2.5 days) and the normothermic periods between torpor bouts may last for days.

### Afrotheria

Heterothermic afrotherians include the tenrecs and golden moles (Tenrecoidea), the elephant shrews (Macroscelidea) and the aardvark (Tubulidentata), which enters shallow torpor during drought (McKechnie and Mzilikazi, 2011; Weyer et al., 2017). Multiday torpor has been observed in the Tenrecoidea and TBDs up to ∼2 days in the Macroscelidea. Arguably one of the most unusual patterns of hibernation known is that of the tenrec, *Tenrec ecaudatus*, in subtropical Madagascar. Tenrecs hibernated underground without periodic arousals for up to 9 months, including the summer, with *T*_b_s>22°C and tracking *T*_soil_ (Lovegrove et al., 2014). Perhaps this species also belongs to the species that can hibernate for one year, but since hibernation was disturbed, this remains to be determined. For the golden mole (*Amblysomus hottentottus longiceps*) in the Drakensberg Mountains of South Africa, data are available only on a single individual and these are very different from the tenrecs. In spring the mole expressed multiday torpor bouts of ∼5 days with *T*_b_ as low as 8.6°C, interrupted by multiday normothermic periods (Scantlebury et al., 2008). Elephant shrews are often considered to be daily heterotherms, however, captive rock elephant shrews (*Elephantulus edwardii*) remained torpid for up to 44 h with minimum *T*_b_ of 9.2°C (Geiser and Mzilikazi, 2011). In the wild, the temporal patterns of torpor in elephant shrews suggest mainly, but not exclusively, daily arousals (Mzilikazi and Lovegrove, 2004) and this is covered below.

### Xenarthra

The pygmy armadillo, *Zaedius pichiy*, hibernates from autumn to winter, with torpor bouts lasting for up to 4.5 days (Superina and Boily, 2007). After the hibernation season, pichis continued to show large daily variation in *T*_b_ until spring.

### Bears and Other Carnivores

Black bears (*Ursus americanus*) maintained under outdoor conditions in Alaska hibernated from November/December to April (Toien et al., 2011). Unlike in other hibernators *T*_b_ in bears fell only to about 30°C, but MR was substantially reduced beyond what is expected from temperature-effects demonstrating a strong metabolic inhibition, but from a rather low normothermic MR because of their large size (Toien et al., 2011). Free-ranging brown bears (*U. arctos*) in Sweden reduced activity, *T*_b_ and heart rate weeks before they began denning (Evans et al., 2016). Bears entered dens around October/November when *T*_a_ was ∼0°C and snow fell and finished denning in early April. During hibernation *T*_b_ fell from ∼38 to 33°C and heart rate from ∼70 to 15 beats/per minute. Bears do not eat, drink or defecate during hibernation, but females may give birth and suckle young. Other carnivores that can show multiday torpor bouts occasionally are European badgers (*Meles meles*), but available information suggests that torpor is restricted to winter (Fowler and Racey, 1988).

### Monotremes

The only known hibernator in the egg-laying mammals is the short-beaked echidna (*Tachyglossus aculeatus*). It is distributed all over Australia and southern Papua and its seasonal expression of torpor differs accordingly (Nicol and Andersen, 1996). In Tasmania and at high elevations in Kosciuszko NP in south-eastern Australia, the hibernation season lasts for up to 10 months from February/March/April to October/November (i.e., austral summer to spring, Table 1; Grigg et al., 1989; Nicol and Andersen, 2007), it is somewhat shorter ∼5 months in the northern Tablelands of NSW (Falkenstein et al., 2001), whereas in Western Australia and Kangaroo island it lasts for 1–3 months (Nicol and Andersen, 1996). Even in the hot and dry climate of south-western Queensland they show torpor bouts of up to 9 days in winter and up to 1 day in summer (Brice et al., 2002). Although in non-reproducing individuals the hibernation season may continue into late spring, reproductively active individuals terminate hibernation in mid-winter (Nicol and Andersen, 2007) and males, which maintain large testes during hibernation, may mate with hibernating females, or with females that aroused from torpor, and, after mating, females expressed multiday, deep torpor (Morrow and Nicol, 2009).

### Marsupials

The Monito-del-Monte (*Dromiciops australis*) a south-American opossum (Didelphidae) hibernates (Bozinovic et al., 2004) apparently mainly in winter. More information on seasonal torpor expression is available for the Australian pygmy-possums (Burramyidae). The mountain pygmy-possum (*Burramys parvus*) is an endangered species limited to high altitudes of the Australian Alps (Broome et al., 2012). It hibernates under snow-covered boulder fields from April/May till September or October (austral autumn to spring, Table 1) depending on the disappearance of snow cover, and males typically terminate hibernation earlier likely to prepare for reproduction (Körtner and Geiser, 1998). In captivity *B. parvus* reduce activity to enhance fattening during the pre-hibernations season and can almost double their body mass during that time (Körtner and Geiser, 1995). Other pygmy-possums are less seasonal in their expression of torpor. In free-ranging eastern pygmy-possums (*Cercartetus nanus*) in a warm-temperate habitat north of Sydney, multiday torpor of up to 20 days was expressed in winter and short bouts of torpor in summer (Turner et al., 2012a). Observational data suggest that torpor in *C. nanus* occurs in all seasons including early summer, but not in late summer to mid-winter, when banksia trees (*Banksia integrifolia*), a major source of nectar, are flowering and animals reproduce (Bladon et al., 2002). As stated above, captive *C. nanus* can hibernate for up to 1 year (Geiser, 2007) and can do so despite expression of short bouts of torpor lasting less than 1 day at the beginning of the hibernation season likely because even during these brief bouts it can reduce TMR to extremely low values of hibernators (Song et al., 1997) like the *Nyctophilus* bats (see above). These data provide further support for the view that these brief torpor bouts are functionally short bouts of hibernation. Western pygmy-possums (*C. concinnus*) in a Mediterranean climate hibernated in winter expressing both brief and multiday torpor bouts of up to 8 days (Turner et al., 2012b). This species was not examined in summer in the field, but captive *C. concinnus* as well as little pygmy-possums (*C. lepidus*) expressed spontaneous torpor throughout the year, and, when held at a constant *T*_a_ of 20°C and natural photoperiod, TBD was affected by photoperiod in *C. nanus* and *C. concinnus* (Turner and Geiser, 2017).

### Birds

The only known avian hibernator is the American poorwill (*Phalaenoptilus nuttallii*). Poorwills breed in the western United States and southern Canada and migrate to the southern United States and Mexico in winter where they hibernate (Brigham, 1992; Woods et al., 2019). Data on free-ranging birds from Arizona show that they entered torpor frequently in winter, often at the base of *Opuntia* cacti, but on sunny days *T*_skin_ fluctuated by >25°C due to passive rewarming by the sun. When birds were artificially shaded they remained inactive for up to 45 days and displayed torpor bouts of 4–7 days, with *T*_skin_ falling below 5°C (Woods et al., 2019). Qualitatively this pattern of torpor expression is similar to that observed on fat-tailed lemurs (Dausmann, 2014; Dausmann and Warnecke, 2016), although birds and mammals have been phylogenetically separated for ∼300 Million years. In summer in southern Canada, poorwills enter short bouts of torpor (up to 36 h) regularly in spring and autumn but less frequently when incubating and only during inclement weather (Kissner and Brigham, 1993).

## Daily Torpor

Daily torpor, unlike hibernation, has been more recently discovered (e.g., Bartholomew et al., 1957; MacMillen, 1965; Morhardt and Hudson, 1966; Dawson and Fisher, 1969). Daily torpor is not as obvious as hibernation because animals often forage daily and without physiological measurements it is difficult to ascertain whether an animal is torpid or simply resting or asleep. Overall, data on seasonal torpor expression especially in free-ranging daily heterotherms are rare.

### Mammals

### Rodents

**FIGURE 3:**
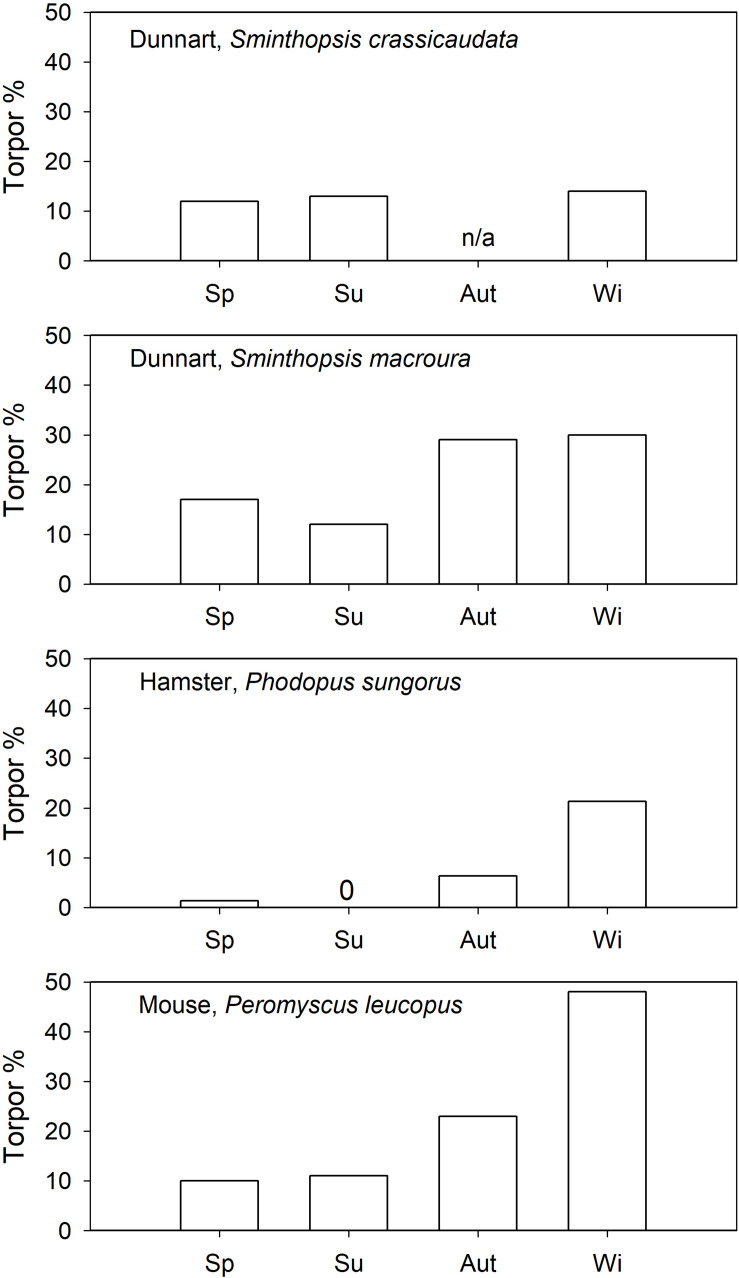
Spontaneous torpor (food *ad libitum*) expression (% of observation days) as a function of season in daily heterotherms. References are provided in the text. 0 = no torpor observed, n/a = data are not available for this season.

The seasonality of daily torpor has been investigated in captive Siberian hamsters, *Phodopus sungorus* (Heldmaier and Steinlechner, 1981), and the data suggested that their expression of torpor is strongly seasonal. Spontaneous torpor (food *ad libitum*) in outdoor enclosures was used from October to March (∼21% of days in winter) but not in summer (Figure 3). Similarly, when held under short photoperiod *P. sungorus* expressed spontaneous torpor, but not under long photoperiod (Geiser et al., 2013). However, torpor could be induced in a summer-acclimated congener, the desert hamster, *P. roborovskii* (Chi et al., 2016). Although it has been suggested that induced torpor and spontaneous torpor in *P. sungorus* differ functionally (Diedrich et al., 2012), some *Phodopus* species obviously have the ability to enter and arouse from torpor even when summer acclimated.

**FIGURE 4:**
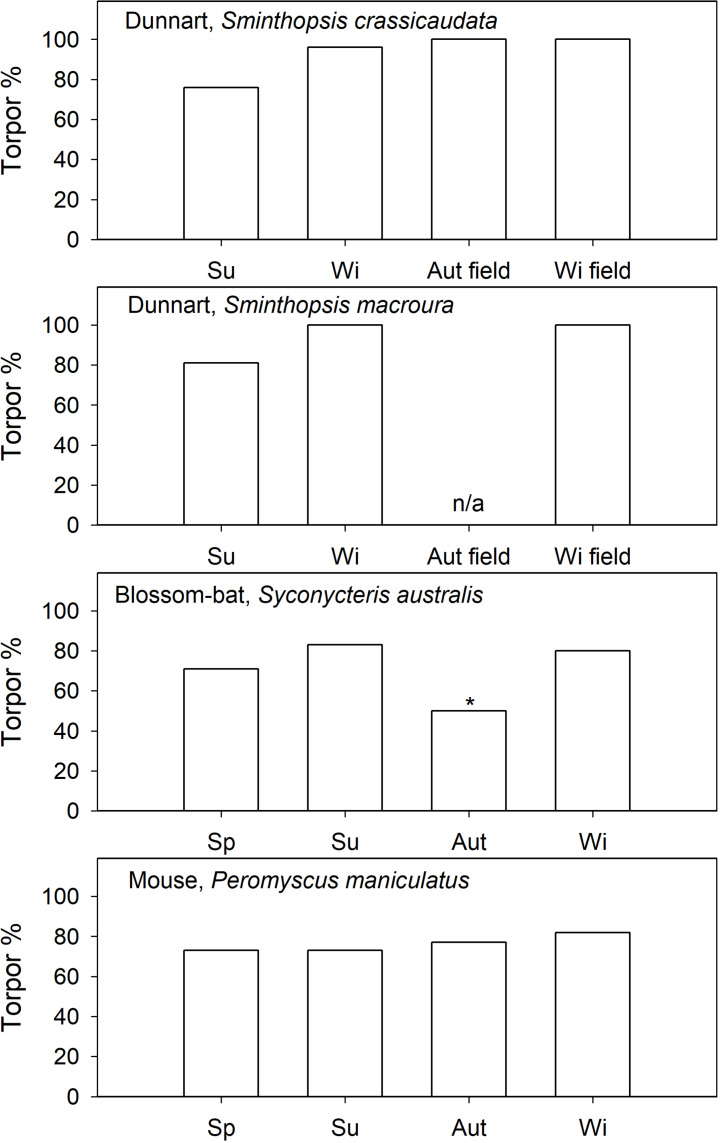
Induced torpor (food restricted) expression (% of observation days) in captive and natural torpor in free-ranging (field) daily heterotherms as a function of season. References are provided in the text. In blossom-bats it is known that they use torpor in autumn, but the % torpor is not available, therefore 50% (*) is assumed. For *S. macroura* field data in autumn are not available.

In north-American “mice” (*Peromyscus* spp.), also of the cricetid family, torpor has been investigated in outdoor cages (Lynch et al., 1978; Tannenbaum and Pivorun, 1988). Spontaneous torpor in *P. leucopus* was observed in all seasons (Figure 3; Lynch et al., 1978), or in all seasons except summer (Tannenbaum and Pivorun, 1988). The torpor incidence in *P. leucopus* increased substantially to >30% for all seasons by food withdrawal. Similarly, *P. maniculatus* expressed spontaneous torpor in autumn (∼4%) and winter (∼10%) but not in spring; food withdrawal (Figure 4) increased the torpor incidence to 70–78% for all four seasons (Tannenbaum and Pivorun, 1989). Torpor duration and depth for *P. maniculatus* were also similar for all seasons investigated (Tannenbaum and Pivorun, 1988), suggesting little or no seasonal functional change.

### Afrotherians

Free-ranging elephant shrews, *Elephantulus myurus*, in KwaZulu Natal, South Africa, expressed torpor throughout the year, but torpor was most pronounced in winter and spring when TBD was ∼8–14 h and *T*_b_ fell to ∼15°C (Mzilikazi and Lovegrove, 2004). However, the maximum TBD of *E. myurus* was 39 h in spring and the minimum *T*_b_ 7.5°C in winter, and in captive *E. edwardii* TBDs of almost 2 days and a minimum *T*_b_ of 9.2°C, likely a regulated value, were observed. These observations suggest that some elephant shrews may be capable of multiday torpor (see above).

### Marsupials

Captive arid zone insectivorous/carnivorous marsupials (*Sminthopsis* spp. and *Dasyuroides byrnei*) held in outdoor enclosures also displayed daily torpor throughout the year (Geiser and Baudinette, 1987). The use of spontaneous (food *ad libitum*) torpor was reduced from 15 to 30% in winter to ∼12% in summer (Figure 3). Occurrence of induced torpor by withdrawal of food and water showed small seasonal changes in *Sminthopsis* spp. with torpor use increasing from ∼75% in summer to 100% of days in winter (Figure 4); the latter is the torpor expression for free-ranging individuals in autumn/winter (Warnecke et al., 2008; Körtner and Geiser, 2009). However, *Sminthopsis* spp. did show seasonal changes in thermal energetics with a 2–3°C reduction in the minimum regulated *T*_b_ and a 30–40% reduction of the minimum TMR from summer to winter (Geiser and Baudinette, 1987; Geiser, 2007), demonstrating, that although animals can use torpor throughout the year, there is a functional seasonal change in physiology.

The kowari (*Dasycercus blythi/cristicauda*) inhabits sandy and stony deserts in inland Australia. It also changed its expression of torpor with season during the cold season, but unlike in many other species this mainly reflected sex and reproductive state rather than weather or habitat (Körtner et al., 2016). Males expressed torpor after the mating season, females mainly during pregnancy, but not during lactation, and TBD in females was almost twice as long as in males (Körtner et al., 2008, 2016).

Most marsupials of the genus *Antechinus* are forest dwelling. Captive brown antechinus (*A. stuartii*) and yellow-footed antechinus (*A. flavipes*) did not express spontaneous torpor in summer, but occasionally did so in winter. Food withdrawal increased daily torpor expression to about 30–80% from autumn to spring when juveniles are excluded, which did express torpor in summer (Geiser, 1988). In the field, *A. flavipes* expressed torpor in winter, but torpor patterns were strongly affected by reproductive status (Parker et al., 2019), whereas in *A. stuartii*, daily torpor in winter was mainly affected by weather (Hume et al., 2020).

Daily torpor in free-ranging sugar gliders (*Petaurus breviceps*) was observed between autumn and spring and mainly on cold, wet winter days (Körtner and Geiser, 2000b). However, these gliders also expressed daily torpor during a category one cyclone with heavy rainfall in a subtropical area in spring (Nowack et al., 2015).

### Birds

### Caprimulgiformes

In Australian tawny frogmouths (*Podargus strigoides*), the largest bird known to use torpor, daily torpor was mainly observed on cold winter nights and mornings, and rarely in autumn and spring, summer data are not available. Frogmouths often entered a night torpor bout followed by endogenous rewarming and re-positioning to new roost with camouflaging background to re-enter a morning torpor bout, which was usually terminated by partial passive rewarming in the sun (Körtner et al., 2000, 2001). No torpor occurred during the spring reproductive season between late September and December (Körtner et al., 2001). In owlet-nightjars (*Aegotheles cristatus*) torpor was used at dawn between late autumn and early spring, but not during other times of the year (Brigham et al., 2000). Winter torpor in arid zone owlet-nightjars was much more pronounced during a drought year (Doucette et al., 2012) and birds roosting in trees expressed torpor about twice as often as those in rock crevices (Doucette et al., 2011). Whip-poorwills (*Caprimulgus vociferous*) rarely used torpor in spring or autumn and not in summer (Lane et al., 2004), and it is uncertain what they do during migration to the south.

### Apodiformes

Andean Hillstars (*Oreotrochilus estella*) in the Peruvian Andes at ∼4000 m elevation used nocturnal torpor both in winter and summer, but winter torpor was more frequent and longer with extremely low minimum *T*_b_s near 7°C (Carpenter, 1974). Although data on the other seasons are not available it is highly likely that they express torpor throughout the year. Captive Rufous hummingbirds (*Selasphorus rufus*) used torpor from spring to autumn. Rather unusual for daily heterotherms, during pre-migratory fattening in autumn when birds were fat for migration, torpor was most pronounced (Hiebert, 1993). Although winter data are not available because the birds migrate south it is likely that they express torpor throughout the year.

### Passeriformes

Alaskan black-capped chickadees (*Poecile atricapilla*) reduced MR during nocturnal torpor to a similar extent in both summer and winter (Sharbaugh, 2001), unlike in the same species and in willow tits (*Parus montanus*) measured at lower latitudes, which expressed shallow torpor only in winter but not in summer (Cooper and Swanson, 1994). In wintering blue tits (*Cyanistes caeruleus*) *T*_b_ fluctuations were affected by *T*_a_ with higher *T*_b_ in experimentally heated tits (Nord et al., 2011), which may explain the differences among populations. Captive passerine sunbirds (*Nectarina famosa*) from South Africa enter nocturnal torpor in summer when exposed to low *T*_a_ (Downs and Brown, 2002) suggesting that torpor may also be used at other times of the year. Free-ranging noisy miner (*Manorina melanocephala*) expressed frequent, shallow nocturnal torpor from autumn to early spring (Geiser, 2019) and fairy wrens (*Malurus cyaneus*) in winter, other seasons were not examined (Romano et al., 2019).

### “Reversed” Seasonal Torpor Expression

Seasonal changes in the patterns of torpor by nectarivorous blossom-bat *Syconycteris australis* (Megachiroptera) from the subtropical east coast of NSW, Australia, were the opposite of those observed for insectivorous microbats and many other heterothermic mammals, which often display more frequent and more pronounced torpor in winter than in summer. Although induced torpor occurrence was similar in summer and winter (Figure 4), average TBD of *S. australis* captured in winter was short (5.5 h) and torpor was shallow with a minimum *T*_b_ of ∼23°C, whereas in bats captured in summer torpor was deep (minimum *T*_b_∼19°C) and long at 7.3 h on average (Coburn and Geiser, 1998). The unusual seasonal response seems to be explained by different day length and food availability. In winter, *T*_a_ on the subtropical east coast is relatively mild and bats can forage for prolonged periods during long nights and have access to an abundance of flowering plants (Coburn and Geiser, 1998). In summer, nights and thus foraging times are brief and the availability of nectar is substantially reduced (Coburn and Geiser, 1998). Thus, the unusual seasonal pattern of torpor use in *S. australis* appears to be an appropriate physiological adaptation to ecological constraints of their subtropical habitat, but it does suggest a seasonal change in physiology.

For an unrelated heterothermic rodent from the Dead Sea desert region in Israel, similar observations have been made. Spiny mice (*Acomys russatus*) in outdoor enclosures under natural food availability, expressed about twice as many torpor bouts and, on average spent about twice the time in torpor in summer (∼780 min) than in winter (∼370 min) to conserve water (Levy et al., 2011). Even when food was offered *ad libitum*, summer torpor was more frequent and longer than winter torpor.

### Seasonal Comparison

**FIGURE 5:**
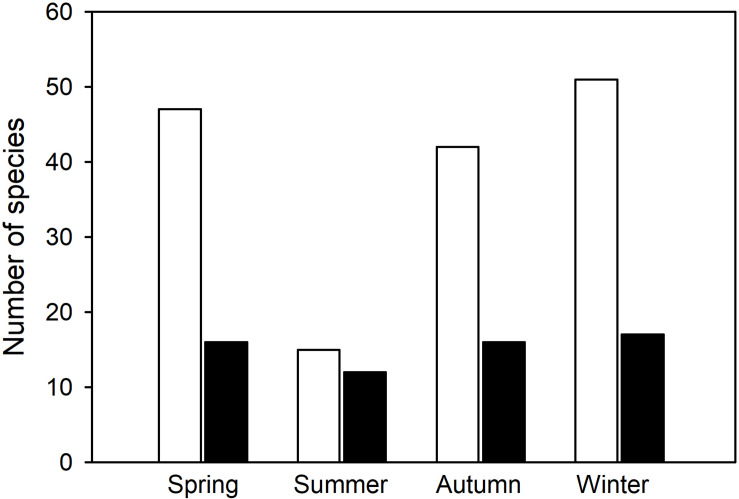
Number of species known to express torpor during different seasons of the year (from Tables 1, 2). In hibernators (white bars), the number of species using torpor changed significantly with season (Chi Square = 20.5, df = 3, *p* < 0.0001), but summer torpor was still used. In daily heterotherms (black bars) there was no change of species number with season (Chi Square = 0.97, df = 3, *p* = 0.81).

Overall, the number of hibernators using torpor (Figure 5) differed significantly among the four seasons (Chi Square = 20.5, df = 3, *p* < 0.0001). In contrast, the number of daily heterotherms using torpor did not change with season (Chi Square = 0.97, df = 3, *p* = 0.81). This is further support for the view that there are two major groups of heterotherms suggesting that not only physiological variables, but also the seasonal expression of torpor differs between most hibernators and daily heterotherms.

**TABLE 2:** The torpor season when daily torpor is used, or is likely to be used, by birds and mammals.

**All year**
Andean Hillstar, *Oreotrochilus estella*, spring and autumn unknown (Carpenter, 1974)
Rufous hummingbird, *Selasphorus rufus*, migratory, winter unknown (Hiebert, 1993)
Sunbird, *Nectarina famosa*, summer (Downs and Brown, 2002)
Black-capped chickadee, *Poecile atricapilla*, spring and autumn unknown (Sharbaugh, 2001)
Fat-tailed dunnart, *Sminthopsis crassicaudata* (Geiser and Baudinette, 1987; Warnecke et al., 2008)
Stripe-faced dunnart, *Sminthopsis macroura* (Geiser and Baudinette, 1987; Körtner and Geiser, 2009)
Kowari, *Dasyuroides byrnei* (Geiser and Baudinette, 1987)
Blossom-bat, *Syconycteris australis* (Geiser et al., 1996; Coburn and Geiser, 1998)
Elephant shrew, *Elephantulus myurus* (Mzilikazi and Lovegrove, 2004)
White-footed mouse, *Peromyscus leucopus* (Lynch et al., 1978)
Deer mouse, *Peromyscus maniculatus* (Tannenbaum and Pivorun, 1989)
Desert hamster, *Phodopus roborovskii* (Chi et al., 2016; Geiser et al., 2019)
Spiny mouse, *Acomys russatus* (Levy et al., 2011)
**Autumn to spring**
Tawny frogmouth, *Podargus strigoides* (Körtner et al., 2001)
Owlet-nightjar, *Aegotheles cristatus* (Brigham et al., 2000)
Whip-poorwill, *Caprimulgus vociferous*, migratory, winter unknown (Lane et al., 2004)
Noisy miner, *Manorina melanocephala* (Geiser, 2019)
Brown antechinus, *Antechinus stuartii* (Geiser, 1988; Hume et al., 2020)
Yellow-footed antechinus, *Antechinus flavipes* (Geiser, 1988; Parker et al., 2019)
Sugar glider, *Petaurus breviceps* (Körtner and Geiser, 2000a; Nowack et al., 2015)
Djungarian hamster, *Phodopus sungorus* (Heldmaier and Steinlechner, 1981)

### What Causes the Seasonal Change in Torpor Expression?

In many species, acclimation or acclimatization to photoperiod or specifically short photoperiod is a strong signal for the preparation for hibernation or the expression of daily torpor. For the hibernators these include dormice, *G. glis* (Morrison, 1964) and European hamsters, *Cricetus cricetus* (Canguilhem et al., 1988). However, more work has been conducted on daily heterotherms such as *Phodopus sungorus* or *Peromyscus maniculatus*, which tend to be highly photoperiodic and respond strongly to exposure to short photoperiod, but low *T*_a_ can amplify or accelerate the response in *P. sungorus* (Lynch et al., 1978; Steinlechner et al., 1986; Tannenbaum and Pivorun, 1988; Geiser and Heldmaier, 1995; Hiebert et al., 2003). In *P. sungorus* pelage color, morphology, thermal and reproductive physiology, and tissue fatty acid composition change in response to photoperiod acclimation, but, with regard to seasonal expression of daily torpor, it is especially spontaneous torpor use that shows the strongest response (see above).

In contrast, in many of the sciurid ground squirrels, which are often viewed as “obligate” hibernators, torpor expression is strongly seasonal and highly predictable. Ground squirrels (*Ictidomys tridecemlineatus*), unlike dormice (*G. glis*) held under the same environmental conditions, did not change torpor expression according to photoperiod (Morrison, 1964). Thus in this and other sciurids, the seasonal use of torpor can be more or less independent of photoperiod and to some extent even from *T*_a_ and is governed principally by a circannual rhythm (Morrison, 1964; Wang, 1978; Kenagy and Barnes, 1988; Barnes, 1996; Geiser et al., 1990; Michener, 1992; Arnold, 1993; Körtner and Geiser, 2000a; French, 2008; Williams et al., 2017).

In species from low latitudes, such as the subtropical blossom-bat (*Syconycteris australis*) although they may show a strong “reversed” seasonal change in torpor expression when captured in different seasons from the field (see above), photoperiod acclimation in captive individuals did not show a strong effect (Geiser et al., 2005). This suggests that other seasonal signals must be used in the wild. Similarly, the mountain pygmy-possum (*Burramys parvus*), which shows seasonal hibernation in the wild (see above), maintained activity and body mass cycles only within the first winter in captivity (Körtner and Geiser, 1995). Despite maintenance under a mimicked “natural” yearly *T*_a_ and photoperiod cycle, this seasonal rhythmicity was lost in the second year in captivity, which again suggest that other seasonal signals must be used in the wild. Therefore, as for other aspect of seasonal torpor use, the control of its expression differs among species and revealing the responsible cues will require further work.

### The Costs and Benefits of Torpor

Avian and mammalian torpor can be highly effective in reducing energy and water use. However, it has been argued that the energy-conserving value of torpor has been overstated (Boyles et al., 2020) and that torpor should be minimized whenever possible because it entails certain risks. Perceived risks include: (i) a physiological dysfunction perhaps due accumulation of waste products at low *T*_b_, (ii) oxidative stress during periodic rewarming, (iii) negative effects on neuronal tissues or memory, (iv) reduced immuno-competence, (v) sleep deprivation, and (vi) increased predation. Many of these risks have been reported as generally applicable (Humphries et al., 2003), although some were based on observations on the eastern chipmunk (*T. striatus*), a non-representative hibernator (see above). Similar concerns of the costs of torpor have been raised by Boyles et al. (2020), but these authors added a nearly complete absence of behavioral responses during torpor (vii) and the increased likeliness of freezing (viii) to the list.

Let us examine these perceived risks in consideration of the seasonal torpor expression and other available information. It is correct that the reasons for periodic arousal from hibernation are still not fully understood and likely involve some malfunction at low *T*_b_, but these can be easily overcome by periodic rewarming and, although this comes at an energetic cost, energy expenditure during hibernation is still only a fraction of that in normothermic individuals. Moreover, in some species hibernating at high *T*_b_s at around 20–25°C such as tenrecs (Lovegrove et al., 2014), hibernation is possible for months without the need to rewarm. Fat-tailed lemurs rely on passive rewarming (Dausmann, 2014) and bats and other species use passive or partially passive rewarming from torpor and thereby minimize energy costs and also the associated oxidative stress (Currie et al., 2015). If oxidative stress does occur in species that do not use passive rewarming, it does not seem to unduly interfere with their wellbeing because heterotherms tend to live longer than homeotherms (Turbill et al., 2011). The memory loss reported for some species, as for example, during hibernation in ground squirrels (Millesi et al., 2001), remains controversial because hibernating bats do not suffer memory loss (Ruczynski and Siemers, 2011). The reduced immuno-competence is a real concern, but often is counteracted by slowed bacterial growth at low *T*_b_. Unfortunately, this is not the case for the new pathogen *Pseudogymnoascus destructans*, a fungus imported 2008/09 from Eurasia, which causes white-nose syndrome in hibernating North American bats, and resulted in catastrophic population declines in many regions (Warnecke et al., 2012). However, more recently surviving bats have developed some immunity (Frick et al., 2017) like their Eurasian counterparts and it appears that survival rates are now improving (Frank et al., 2019). Sleep deprivation during deep torpor (Daan et al., 1991) also can be counteracted by periodic rewarming and again the main costs seems to be energy expenditure, which, as is stated above, is much lower than in normothermic animals despite endogenous rewarming. The perceived increased predation risk during torpor is based on observations of predation of hibernating individuals such as marmots by badgers (Armitage, 2004) or bats by blue tits (Estok et al., 2009). However, even when badgers did find the marmot colony, predation rate was still <5% (Armitage, 2004) and population studies show that hibernators have much better survival rates during winter hibernation than during the active season in summer (Kawamichi and Kawamichi, 1993; Lebl et al., 2011). Although torpid animals are slower than they are during normothermia, they can move nevertheless, many from around *T*_b_ 15°C (Walhovd, 1979; Rojas et al., 2012) and hibernating bats can move at *T*_b_ as low as 5–8°C (Choi et al., 1998; Bartonička et al., 2017). With regard to exposure to very low *T*_a_, thermoregulation during torpor does have a negative effect on telomere length (Nowack et al., 2019). However, the likeliness of freezing during hibernation is rather low because of selection of appropriate hibernacula sites, and, even in arctic hibernators that hibernate well below 0°C, endogenous heat production during torpor maintains a large *T*_b_–*T*_a_ differential and prevents freezing in most individuals, again at a lower energetic cost than for normothermic individuals (Barnes, 1989; Buck and Barnes, 2000; Richter et al., 2015). Thus, it appears that many of the perceived risks of torpor have been overstated, and the main cost often is a somewhat increased energy expenditure, which is nevertheless much lower than during normothermia. Considering that hibernation is used throughout the year by pygmy-possums, bats and dormice and perhaps other species, and that torpor is used in the presence of food by many species, the view that torpor should be minimized whenever possible is not supported. Instead these and other data on expression of daily torpor for much of the year suggest that the multiple selective advantages of torpor and the diversity of torpor are still not fully appreciated.

## Author Contributions

The author collected the data and wrote the manuscript.

## Conflict of Interest

The author declares that the research was conducted in the absence of any commercial or financial relationships that could be construed as a potential conflict of interest.
